# The Hidden Heart: Exploring Cardiac Damage Post-Stroke: A Narrative Review

**DOI:** 10.3390/medicina60101699

**Published:** 2024-10-16

**Authors:** Marian Mitrică, Lorenzo Lorusso, Alexandru-Andrei Badea, Carmen-Adella Sîrbu, Andreea Pleșa, Ana-Maria Alexandra Stănescu, Florentina Cristina Pleșa, Octavian Mihai Sîrbu, Alice Elena Munteanu

**Affiliations:** 1 Clinical Neurosciences Department, ‘Carol Davila’ University of Medicine and Pharmacy, 050474 Bucharest, Romania; titimitrica@yahoo.com (M.M.); cristina.plesa@umfcd.ro (F.C.P.);; 2 Neurology Unit, Neuroscience Department A.S.S.T. Lecco, Merate Hospital, 23807 Merate, Italy; l.lorusso@asst-lecco.it; 3 Department of Cardiology, ‘Dr. Carol Davila’ Central Military Emergency University Hospital, 010825 Bucharest, Romania; badea.alexandru97@gmail.com (A.-A.B.); dralicepopescu@yahoo.com (A.E.M.); 4 Academy of Romanian Scientists, 050045 Bucharest, Romania; 5 Doctoral School, Faculty of Medicine, “Carol Davila” University of Medicine and Pharmacy, 050474 Bucharest, Romania; andreea.plesa@drd.umfcd.ro; 6 Department No. 5, ‘Carol Davila’ University of Medicine and Pharmacy, 050474 Bucharest, Romania; alexandra.stanescu@umfcd.ro; 7 Department of Medical-Surgical and Prophylactical Disciplines, Faculty of Medicine, ‘Titu Maiorescu’ University, 031593 Bucharest, Romania

**Keywords:** myocardial dysfunction, stroke, brain, catecholamine surge, cardiac dysfunction, cardiac enzymes, neurogenic stunned myocardium, Takotsubo syndrome, neurogenic stress cardiomyopathy, stroke–heart syndrome, autonomic dysfunction

## Abstract

Stroke–heart syndrome (SHS), a critical yet underrecognized condition, encompasses a range of cardiac complications that arise following an ischemic stroke. This narrative review explores the pathophysiology, clinical manifestations, and implications of SHS, focusing on the complex interplay between the brain and the heart. Acute ischemic stroke (AIS) triggers autonomic dysfunction, leading to a surge in catecholamines and subsequent myocardial injury. Our review highlights the five cardinal manifestations of SHS: elevated cardiac troponin (cTn) levels, acute myocardial infarction, left ventricular dysfunction, arrhythmias, and sudden cardiac death. Despite the significant impact of these complications on patient outcomes, there is a notable absence of specific guidelines for their management. Through a comprehensive literature search, we synthesized findings from recent studies to elucidate the mechanisms underlying SHS and identified gaps in the current understanding. Our findings underscore the importance of early detection and multidisciplinary management of cardiac complications post-stroke. Future research should focus on establishing evidence-based protocols to improve clinical outcomes for stroke patients with SHS. Addressing this unmet need will enhance the care of stroke survivors and reduce mortality rates associated with cardiac complications.

## 1. Introduction

Cardiovascular and cerebrovascular diseases remain the foremost causes of mortality and morbidity worldwide, with their incidence steadily increasing annually [1]. According to the American Heart Association (AHA), stroke ranks as the second leading cause of death in the United States, accounting for 17.3% of deaths in 2020, following coronary artery disease (17.3%). From 2011 to 2021, the actual number of stroke deaths increased by 26.3% (from 128 932 to 162 890 deaths) [2]. Cardiovascular complications are the second most common cause of mortality after neurological damage in acute ischemic stroke (AIS) patients [3]. Cardiac injury significantly impacts both the acute and long-term survival of stroke patients. A study including 360,000 patients with AIS showed that approximately 27% developed cardiovascular complications within the first four weeks post-stroke [4]. Compared with individuals without a stroke, this group has a 25 times higher risk of developing major cardiovascular conditions [5]. The most critical complications of ischemic stroke occur acutely within the first three months, affecting 4–19% of patients [6]. Furthermore, heart disease is a substantial risk factor for ischemic stroke, as evidenced by the Framingham study, which indicated that the risk of stroke doubles in patients with coronary artery disease, triples in those with hypertension, quadruples in individuals with heart failure, and increases fivefold in those with atrial fibrillation (AF) [7]. Despite existing guidelines for managing cardiovascular risk post-stroke, there is a significant lack of guidelines explicitly addressing the management of post-stroke cardiovascular complications from a cardiological perspective.

The concept of brain–heart syndrome was first introduced in 1947 by Byer et al., who identified cerebrovascular disease as a cause of myocardial injury and certain rhythm disorders [8]. Currently, brain–heart syndrome is defined as a regional wall motion abnormality of the ventricle due to central nervous system disease. Cerebrovascular diseases that can be included in this syndrome such as ischemic stroke, subarachnoid hemorrhage, cerebral hemorrhage, infectious meningitis, brain trauma, and central sleep apnea syndrome [9].

Cardiac injury refers to damage to the heart muscle caused by factors like reduced blood flow (ischemia), inflammation (myocarditis), physical trauma, toxins (drugs, alcohol), or chronic conditions (hypertension). It is often detected through symptoms, biomarkers (cardiac enzymes), or imaging studies. Cardiac injury as a complication of cerebrovascular disease is termed brain–heart syndrome, while cardiac injury secondary to AIS is referred to as stroke–heart syndrome (SHS). SHS includes five categories of cardiac manifestations: elevated cardiac enzyme levels secondary to ischemic or non-ischemic myocardial injury, typically asymptomatic; acute myocardial infarction; left ventricular dysfunction; heart failure and Takotsubo syndrome; sudden cardiac death post-stroke; and electrocardiogram (ECG) abnormalities and arrhythmias [10]. The incidence of these complications ranges from 3% for myocardial infarction to at least 50% for newly appearing ECG changes [11]. These cardiac manifestations are classified under SHS if they occur within the first 30 days post-stroke; beyond this period, they are considered long-term sequelae. They may present as isolated or may overlap in the individual patient, often without pre-existing structural or functional cardiac disease, usually manifesting within 72 h post-stroke, and tend to be transient, improving as the primary neurological condition ameliorates over subsequent weeks [12].

The objective of this article is to conduct a comprehensive review of the current literature, offering a coherent description of the intricate relationship between myocardial dysfunction and stroke. This narrative review delves into the pathophysiology and clinical manifestations of SHS. Additionally, it discusses the current management of SHS manifestations, and emphasizes the critical need for the development of evidence-based guidelines to effectively manage and monitor stroke patients who present with post-stroke cardiac injury, ensuring optimal clinical outcomes and long-term care.

## 2. Materials and Methods

This article is a narrative review that synthesizes the existing literature on myocardial dysfunction following stroke. A comprehensive literature search was conducted using the electronic databases PubMed, Google Scholar, and ScienceDirect. The search strategy employed keywords and phrases such as “myocardial dysfunction”, “stroke”, “catecholamine surge”, “cardiac dysfunction”, “cardiac enzymes”, “neurogenic stunned myocardium”, “Takotsubo syndrome”, “neurogenic stress cardiomyopathy”, and “stroke–heart syndrome”. This approach enabled the identification of relevant studies that contributed to the understanding of myocardial dysfunction after a stroke.

Studies were selected based on several inclusion criteria: articles had to be published in English, with dates ranging from January 2015 to July 2024. The selection process focused on peer-reviewed journal articles, clinical trials, reviews, and meta-analyses that directly addressed the pathophysiology, incidence, and clinical outcomes of myocardial dysfunction post-stroke. Exclusion criteria included non-English articles, conference abstracts, and studies primarily focused on non-cardiac complications of stroke.

Data were extracted from the included studies, and the gathered information was qualitatively analyzed to identify recurring themes, patterns, and gaps in the current understanding of myocardial dysfunction after a stroke. The findings were systematically organized into sections that explore the catecholamine surge, blood–brain barrier dysfunction, inflammation, gut microbiome, and related cardiac complications such as neurogenic stress cardiomyopathy. This structured approach facilitated a comprehensive review of the existing literature and provided a coherent framework for understanding the complexities of myocardial dysfunction in the context of stroke.

## 3. Pathophysiology of Stroke–Heart Syndrome

**Figure 1:**
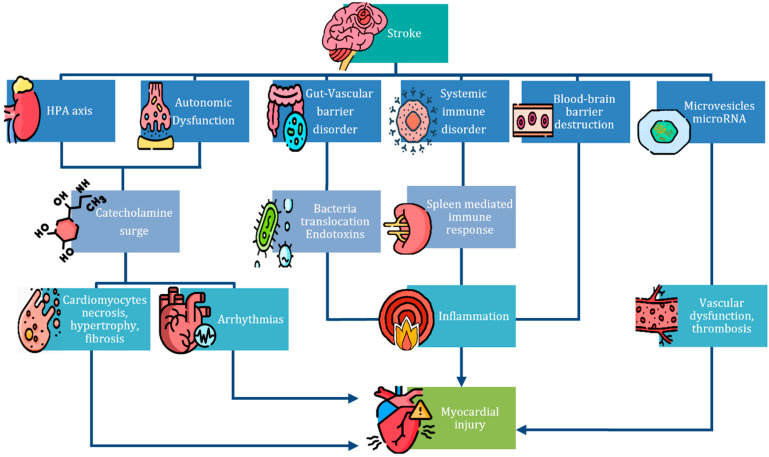
Pathophysiology of SHS [8,13]. Legend: this picture summarizes the mechanisms underlying SHS, offering a visual representation of the various factors that contribute to cardiac injury following a stroke.

SHS refers to the intricate interplay between cerebrovascular accidents and cardiac complications. The pathophysiology of this syndrome is complex and multifactorial, involving both direct and indirect mechanisms that contribute to cardiac injury following a stroke. One of the central hypotheses is the catecholamine surge theory, which posits that a stroke triggers a significant release of catecholamines due to central autonomic dysfunction. This autonomic disruption, coupled with a robust inflammatory response, forms the basis of SHS, leading to cardiac complications ranging from arrhythmias to myocardial injury (Figure 1).

## 4. The Catecholamine Surge Theory

It is currently hypothesized that the primary mechanism underlying SHS involves central autonomic dysfunction, with a contributory role played by the inflammatory response [13]. Key brain regions, including the insular cortex, prefrontal cortex, cingulate cortex, amygdala, hypothalamus, and hippocampus primarily govern the regulation of autonomic activity [14]. Acute injury to the central autonomic nervous system following an ischemic stroke results in the dysfunction of the sympathetic and parasympathetic networks responsible for transmitting impulses from the brain to the heart. Consequently, myocardial nerve endings release substantial quantities of catecholamines (adrenaline, noradrenaline, and dopamine), leading to excessive increases in contractility, myocardial hypertrophy, and impaired relaxation [15]. The concentration of circulating catecholamines during a stroke is associated with an elevated risk of myocardial injury. A chronic increase in circulating catecholamines promotes inflammation, fibrosis, and contraction band necrosis [16]. Contraction band necrosis is a form of uncontrolled cell death specific to myocardial cells, arising from hypercontractility due to excessive adrenergic stimulation. Recent studies demonstrated that enhanced sympathetic nervous system activity induces pro-inflammatory monocytosis in stroke. Acute stress, such as stroke, elevates bone marrow levels of noradrenaline, consequently diminishing the expression of hematopoietic stem cell retaining factors in bone marrow stromal cells through β2-receptor signaling [17]. Thus, catecholamines induce hematopoietic stem cell mobilization, enhancing hematopoiesis with a predominance of myeloid lineage [18]. Nevertheless, innate immune cells express α2- and β2-adrenergic receptors with pro- and anti-inflammatory effects, respectively. Studies show that catecholamines function as immunomodulators, and can be immunosuppressive, pro-inflammatory, or both at the same time, depending on the way myeloid cells process these signals according to the context and concentration of catecholamines [19,20].

On the other hand, immune cells locally produce and release catecholamines with autocrine and paracrine effects, thus contributing to the excessive sympathetic stimulation [21].

Catecholamines activate cardiac β-adrenergic receptors, which are coupled with stimulatory G proteins that, in turn, activate adenylate cyclase, leading to increased intracellular production of cAMP. This elevation in cAMP stimulates protein kinase A, which phosphorylates L-type sarcolemmal calcium channels, facilitating calcium influx into the cytosol. Additionally, protein kinase A phosphorylates phospholamban, enhancing the activity of calcium channels in the sarcoplasmic reticulum, thereby releasing calcium into the cytosol. Excessive cytosolic calcium release depletes adenosine triphosphate (ATP) and overflows mitochondria with calcium, increasing mitochondrial oxidative stress. This oxidative stress increases mitochondrial membrane permeability, releasing reactive oxygen species into the cytosol and reducing mitochondrial ATP production. Consequently, myocardial cell damage may be reversible or lead to cell death through myocytolysis [15].

Conversely, a stroke represents a significant acute stressor for the organism. In response to such a stressor, the body’s physiological reaction encompasses both rapid and delayed responses [22]. The rapid response is facilitated by the activation of the sympathetic–adrenomedullary (SAM) axis, resulting in the secretion of adrenaline and noradrenaline from the adrenal medulla, and the release of noradrenaline from sympathetic nerve terminals. The slow response is governed by the hypothalamic–pituitary–adrenal (HPA) axis. This involves the stimulation of the hypothalamic paraventricular nucleus, which is the central component of the HPA axis, to release corticotropin-releasing hormone (CRH) and vasopressin. These hormones, in turn, prompt the anterior pituitary to secrete adrenocorticotropic hormone (ACTH), which subsequently stimulates the adrenal cortex to produce and release cortisol [22]. The excessive release of cortisol exerts cardiotoxic effects through its pro-inflammatory properties and by enhancing the effects of catecholamines. A correlation has been observed between serum cortisol levels and the severity of cerebral injury, along with mortality rates, following stroke [23].

The cardiac complications resulting from brain injury can vary depending on the location of the injury. The insula cortex influences multiple autonomic functions through its connections with sympathetic preganglionic areas. Due to its proximity to the middle cerebral arteries, the insular cortex is particularly susceptible to cerebrovascular injuries. The impact of an insular injury and the associated degree of autonomic dysfunction differs based on the affected region. Damage to the right insular cortex or other regions in the right hemisphere, such as the basal ganglia, parietal cortex, frontal cortex, thalamus, or amygdala, leads to increased sympathetic activity, whereas damage to the left insular cortex increases parasympathetic activity [24]. Alterations in autonomic activity result in variations in blood pressure (e.g., non-dipper or riser patterns) and heart rate, along with sleep-related breathing disorders, cardiac arrhythmias, myocardial ischemia, and ventricular dysfunction, often accompanied by elevated levels of specific myocardial injury biomarkers such as serum N-terminal pro-B-type natriuretic peptide (NT-proBNP) and cardiac troponin (cTn) [25]. Studies indicate that a right insular lesion is associated with a higher risk of cardiac complications and increased long-term mortality [26].

Furthermore, as a consequence of the functional deficits resulting from stroke, patients may develop an anxiety depressive disorder characterized by a chronic response to psycho-emotional stress. Post-stroke depression affects approximately 85% of stroke patients, leading to more severe functional impairments, greater challenges in rehabilitation, and increased social isolation [27]. Anxiety and severe depression are also recognized as non-traditional risk factors for coronary heart disease and heart failure [28]. Patients with cardiovascular diseases are also susceptible to comorbid depression. This creates a vicious cycle involving cerebral injury, cardiac injury, and depressive disorder, all of which are associated with chronic excess cortisol and chronic systemic inflammation [29,30].

## 5. Blood–Brain Barrier and Inflammation

The blood–brain barrier (BBB), composed of endothelial cells, astrocytes, pericytes, and the basement membrane, is a dynamic structure that regulates the interaction between the brain and the circulatory system. This barrier prevents the penetration of neurotoxins and hydrophilic molecules that may arise from changes in blood composition. Damage to BBB integrity is the initial step in the pathophysiological process of stroke. BBB injury during stroke is caused by ischemia, which directly damages the endothelial cells of the BBB [31].

Following neuronal injury, large amounts of ATP are released extracellularly, activating resident microglia and promoting the local production of pro-inflammatory cytokines (e.g., IL-2, IL-6, myeloperoxidase, integrins), which stimulate the generation of massive amounts of reactive oxygen species. Damaged endothelial and neuronal cells, along with brain-derived antigens, cross the BBB and reach peripheral organs via the circulatory system, triggering a systemic inflammatory response with an increased production of pro-inflammatory cells that damage target organs. Pro-inflammatory cytokines can accumulate in endothelial cells, causing endothelial dysfunction, microvascular spasms, and the destruction of collagen in coronary atherosclerotic plaques, thereby promoting the onset of coronary syndrome.

The spleen plays a significant role in the severity of the immune response by increasing the production of circulating lymphocytes and pro-inflammatory cytokines.

The increased permeability of the BBB permits the entry of inflammatory factors from the periphery, which further compromise BBB integrity and exacerbate ischemia-induced inflammation and neuronal damage. S100b serves as a marker of BBB dysfunction, correlating with the extent of brain injury and the degree of systemic inflammation [32]. S100B has also been identified as an indicator of myocardial injury and ischemia reperfusion injury. The systemic immune response is integral to the SHS mechanism. Systemic inflammation and BBB damage are initiated and sustained by cytokines, chemokines, stress hormones, and the activity of the autonomic nervous system. Systemic inflammatory response syndrome (SIRS) is a complication that occurs in 18–53% of ischemic stroke patients [33]. SIRS is characterized by a body temperature > 38 °C or <36 °C, tachycardia (>90 bpm), tachypnea (>20 breaths per minute or PaCO^2^ < 32 mmHg), leukocytosis (>12,000/mm^3^) or leukopenia (<4000/mm^3^), or >10% immature neutrophils in the peripheral blood [34]. SIRS following a stroke is a critical determinant of stroke severity. Cytokine levels rise acutely after a stroke and can persist for hours or even days. Conversely, cardiac remodeling continues beyond the resolution of the short-term systemic inflammatory response post-stroke. Elevated levels of cytokines in the systemic circulation, particularly TNF-α and IL-1β, have been observed to induce troponin I damage, thereby impairing cardiac contractility [35]. Pro-inflammatory cytokines secreted by glial cells and damaged neurons activate the hypothalamus, leading to an elevation in sympathetic nervous system activity and an increase in catecholamine synthesis [36]. It has been observed that catecholamine release, associated with parasympathetic dysfunction, triggers a systemic inflammatory response, leading to impaired cardiomyocyte function, thrombus formation, and myocardial necrosis.

## 6. Microbiome and Stroke

The gut vascular barrier (GVB) serves as a dynamic interface that restricts the entry of toxins and pathogens from the digestive lumen into the bloodstream while permitting the absorption of water and nutrients. Digestive or extra digestive conditions, along with cardiovascular or cerebrovascular diseases, can disrupt the microbiota composition. In the case of stroke, there is a reduction in beneficial bacteria, favoring opportunistic bacteria that exacerbate systemic inflammation. This occurs by compromising the GVB, increasing its permeability, and allowing the translocation of bacteria and endotoxins into the bloodstream, thereby further amplifying systemic inflammation [37]. The immune response triggered by bacterial translocation is associated with an exacerbation of cardiac dysfunction. It has been observed that the intestinal levels of certain bacteria, such as Proteus, are directly proportional to the severity of the stroke [38].

Metabolites resulting from alterations in the microbiota composition, such as endotoxins, indoxyl sulfate, and trimethylamine-N-oxide, contribute to cardiac damage. Indoxyl sulfate activates the NLRP3 inflammasome, leading to myocardial dysfunction and cardiac remodeling [39]. Trimethylamine-N-oxide enhances platelet activity, thereby increasing the risk of thrombosis and subsequently elevating the risk of myocardial and cerebral infarctions [40].

## 7. Circulating Microvesicles and microRNA

Microvesicles in the blood post-stroke predominantly originate from the vascular endothelium and stimulate endothelial cells to release IL-6, a cytokine with vasoconstrictive effects, including within the heart. Microvesicles derived from platelets and the injured brain alter platelet activity, increasing the risk of thrombosis. MicroRNAs (miRNAs), non-coding RNAs involved in various processes such as inflammation, angiogenesis, and hypoxia response, are also affected following a stroke. Specifically, miR-126 deficiency has been associated with an increased risk of AF, ischemic coronary disease, and heart failure [41]. Stroke reduces the serum and cardiac expression of miR-126, with this deficiency being associated with an increased risk of post-stroke cardiovascular complications [42].

## 8. Manifestations of Stroke–Heart Syndrome

**Figure 2:**
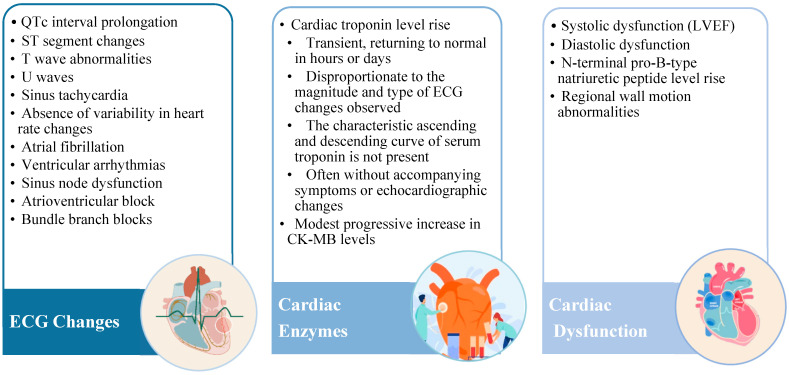
Manifestations of SHS [8,10]. Legends: ECG—electrocardiogram; LVEF—left ventricular ejection fraction.

SHS encompasses a variety of cardiac complications that can occur following a stroke, including ECG changes, cardiac enzyme elevation, and cardiac dysfunction. These manifestations are complex and multifactorial, often involving a combination of autonomic dysfunction, myocardial damage from inflammation and fibrosis, genetic predisposition, and psychological stressors. These manifestations are summarized in Figure 2.

## 9. ECG Changes

The specific mechanism underlying ECG alterations and arrhythmias post-stroke is multifactorial, likely involving autonomic dysfunction and myocardial damage associated with inflammation and fibrosis, shaped by genetic susceptibility and the patient’s psychological and emotional state.

The most common ECG abnormalities include repolarization disorders such as QTc interval prolongation (20–65%), ST segment changes (15–25%), and T wave abnormalities (inverted or high large T waves) (16–40%) [43,44,45]. These abnormalities typically manifest during the acute phase of the stroke, with 85% being detected within the first 24 h, and they generally resolve within the initial days [8,46]. ECG changes, including rhythm disorders, are more prevalent in elderly patients, those with higher stroke severity assessed by the National Institutes of Health Stroke Scale (NIHSS), and individuals with altered myocardial injury marker levels [47,48,49]. The NIHSS measures stroke severity through 15 items, assessing consciousness, language, motor skills, and sensory functions. Scores range from 0 (no impairment) to 42 (severe impairment). ECG abnormalities may precede structural changes detectable by echocardiography, with inverted T waves potentially predicting the presence of a parietal kinetic disorder, particularly when accompanied by elevated serum levels of cTn. Post-stroke ECG changes, such as broad T waves in aVR, QT prolongation, and U waves, are associated with an increased risk of myocardial injury, arrhythmias, stroke recurrence, and mortality [45,50]. The degree of post-stroke disability, as assessed by the modified Rankin scale (mRS), is also greater in patients who exhibit detectable ECG changes [7].

In patients with non-traumatic subarachnoid hemorrhages, it was observed that ECG changes were more prevalent in patients who either did not survive or who presented with a higher risk of mortality, as assessed by a Hunt–Hess score of 4–5 [51].

Cardiac rhythm and conduction disorders occur in 22% of post-stroke patients, with the most frequent being sinus tachycardia (24%), AF (28.1%), ventricular arrhythmias (3.59%), sinus node dysfunction (14%), atrioventricular block (6.4%), bundle branch blocks (16.8%), and other arrhythmias such as atrial flutter [44,52]. It has been observed that ischemic stroke occurring in the right hemisphere, particularly involving the central regions, is more frequently associated with abnormal ECG changes [53,54]. Among rhythm disorders, tachyarrhythmias were detected more frequently (27.1%) compared with bradyarrhythmias (3.9%) [55].

Factors such as the maximum heart rate and the absence of variability in heart rate changes, particularly the lack of nocturnal dipping, have been identified as predictors of a long-term poor prognosis in stroke patients [56].

Tachycardia by itself can lead to either hypoperfusion or hyperperfusion in ischemic brain regions where cerebral autoregulation of blood flow is compromised or absent, thereby exacerbating brain injury [57]. An elevated heart rate may precipitate higher oxidative stress and endothelial dysfunction, both of which are implicated in the pathogenesis of atherosclerosis. Additionally, ventricular dysfunction resulting from sustained tachycardia, reduced coronary perfusion, and renal impairment could further elucidate the correlation between increased heart rate and negative clinical outcomes [58]. While alterations in heart rate can have detrimental effects, they are not invariably caused by brain injury. These changes may also be attributed to other comorbid conditions, including dehydration, infection, hyperthyroidism, or cardiac arrhythmias [59].

Sudden cardiac death can result from fatal or near-fatal arrhythmias, particularly ventricular tachycardia, which may progress to ventricular fibrillation. This risk is especially pronounced in cases of brain lesions affecting the autonomic system’s regulatory centers in the right hemisphere [60] The occurrence of severe arrhythmias is attributable to complex multifactorial mechanisms, including myocardial injury and autonomic dysfunction, influenced by genetic susceptibility, along with the patient’s emotional state and cognitive status [61]. Given the potential for life-threatening complications, clinical guidelines recommend measuring cardiac biomarkers and performing an ECG upon admission for patients with AIS. Additionally, continuous cardiac monitoring should be conducted for at least 24 h following the event [62].

A concept introduced in 2017 and recently incorporated into the 2020 European Society of Cardiology guidelines for AF is AF diagnosed after stroke (AFDAS). This concept aims to differentiate between AF, which is a causative factor preceding ischemic stroke, and AF, which develops as a complication following the stroke [63]. Patients with cerebral injury face a significantly increased risk of subsequently developing AF. This risk is mediated by both cardiogenic and neurogenic factors. The cardiogenic mechanism encompasses pre-existing cardiovascular risk factors and a degree of atrial cardiopathy, which may include previously undiagnosed episodes of AF. The neurogenic mechanism involves structural changes in the left atrium due to systemic inflammation and autonomic dysfunction associated with SHS [10,64]. It has also been demonstrated that emotional stress, along with certain psychiatric conditions such as depressive disorder, can act both as independent risk factors and as triggering factors for AF [65]. The structural substrate of AFDAS is characterized by accelerated fibrosis at the border between the left atrium and the pulmonary veins [66]. In this context, AF may be transient, typically lasting around 23 days with a potential for recurrence, or it may persist or progress to a permanent state [67,68]. AF is a significant cardiovascular complication, associated with a higher likelihood of death, dementia, and stroke recurrence compared with patients without AF [69,70]. Inversely, patients with post-stroke onset of AF generally have a better prognosis compared with those with pre-existing AF. This is attributed to fewer cardiac structural changes, reduced cardiovascular comorbidities, and a lower risk of stroke assessed by the CHA2DS2-VASc score [71]. The CHA2DS2-VASc score is a clinical tool used to estimate the risk of stroke in patients with AF. It assigns points based on the presence of various risk factors: congestive heart failure (1 point), hypertension (1 point), age 75 or older (2 points), diabetes mellitus (1 point), history of stroke or thromboembolism (2 points), vascular disease (1 point), age 65–74 (1 point), female sex (1 point). A score of 0 indicates low risk, 1 indicates moderate risk, and 2 or more suggests high risk.

## 10. Cardiac Enzymes

Due to the potential association between ischemic stroke and ischemic coronary disease, it is recommended to measure cardiac enzymes upon admission for all stroke patients. Creatine kinase (CK) is a crucial enzyme involved in cellular energy metabolism and is widely expressed in various tissues, particularly in muscle and brain tissues. It has tissue-specific distribution, with three isozymes present in the cytoplasm: CK-MM, CK-MB, and CK-BB. CK-MM is predominantly found in skeletal muscle, CK-MB is primarily located in cardiac muscle, and CK-BB is mainly present in the brain. CK-MB is an enzyme that lacks cardiac specificity; therefore, elevated levels in the context of a massive stroke may not necessarily indicate a cardiac cause. The serum level of CK can be increased due to muscle injury, intramuscular injections, chronic kidney disease, intense physical exertion, medications, or toxins. Typically, there is a modest progressive increase in CK-MB levels in AIS, but this increase has not been proven to impact clinical outcomes. Conversely, elevated CK serum concentrations due to brain tissue ischemia and inflammation are associated with higher NIHSS and modified Rankin scale (mRS) severity scores [72].

Troponin is a biomarker with higher specificity and sensitivity for myocardial ischemia. An increase in cTn is considered significant if it exceeds 20% of the upper limit of the reference range. It is an effective diagnostic tool within the first 4–5 h following a stroke [73,74]. Usually, this marker increases transiently and returns to normal in hours or days [75]. Tn release can occur through the following six primary mechanisms (although, clinically, it is challenging to determine the exact one): (1) myocyte necrosis, the most common cause; (2) apoptosis, where caspases are activated to mediate the breakdown of structural proteins during programmed cell death; (3) myocyte turnover, a continuous process of replacement and regeneration throughout life; (4) the cellular release of troponin degradation products without cell death or membrane disruption, allowing small fragments of cTn to pass through an intact membrane; (5) increased cell wall permeability caused by myocardial stretch or ischemia; and (6) the active secretion of vesicles without necrosis [49].

Low levels of elevated troponin can indicate micro-injuries, which are small or repeated heart muscle injuries that occur without symptoms, often due to chronic conditions like hypertension, diabetes, or atherosclerosis. Elevated troponin levels have also been linked to structural heart changes, such as increased left ventricular mass or atrial enlargement, which signal early subclinical cardiac damage. Furthermore, elevated troponin is associated with diastolic dysfunction, even in individuals who have not yet developed overt heart failure.

In approximately one in four cases of AIS, patients are diagnosed with acute myocardial injury. The term “myocardial injury” applies to any patient with a cTn value above the 99th percentile URL, regardless of the underlying cause. Often, the elevation in cTn serum levels occurs without accompanying symptoms or echocardiographic changes. Therefore, measuring cTn serum levels during stroke presentation is imperative [62,76]. Additionally, cardiovascular magnetic resonance (CMR) imaging reveals focal myocardial fibrosis in approximately 30% of patients with AIS, typically presenting with an acute or subacute ischemic pattern [77]. Advanced age, elevated LDL levels, renal insufficiency, ECG changes, and a history of structural or coronary heart disease in stroke patients are associated with a higher likelihood of detecting elevated cTn levels [52]. Elevated cTn levels in stroke patients are associated with an increased severity of cognitive dysfunction, a higher risk of cardiovascular events, and greater mortality from stroke, heart disease, and cancer, observed in the acute phase and up to one year later [78,79,80]. The mortality rate for stroke patients with elevated levels of cTn is six times higher compared with those with normal levels [81]. The cTn level serves as a prognostic biomarker in patients with ischemic stroke, irrespective of the therapeutic approach employed, whether thrombolytic or interventional [82]. Additionally, extremely high levels of cTn are associated with an increased risk of ventricular dysfunction, including reduced left ventricular ejection fraction and segmental ventricular hypokinesia [83].

The level of cTn can also provide insights into the location of the cerebral insult. Damage to the right anterior insular cortex is more frequently associated with sympathetic hyperactivity and secondary acute myocardial injury. An AIS affecting the insular cortex may result in more significant myocardial damage, leading to persistently elevated cTn levels. In contrast, an AIS involving other regions of the autonomic system is typically associated with transient increased levels and less severe cardiac damage [84].

In the case of subarachnoid hemorrhage, an elevated level of cTn in the acute phase may indicate cerebrovascular complications such as vasospasm or delayed cerebral ischemia. Therefore, cTn alone has limited value in predicting the progression of subarachnoid hemorrhage [85].

The mechanism by which SHS is associated with elevated cardiac enzymes involves, on the one hand, cardiotoxic circulating catecholamines that induce myocytolysis, and, on the other hand, coronary microvascular dysfunction that also leads to myocytolysis [86]. The severity of AIS, as indicated by a higher NIHSS score, is correlated with greater activation of the hypothalamic–pituitary–adrenal axis [87]. On the other hand, predisposing factors for stroke—such as hypertension, diabetes, obesity, and dyslipidemia—are also associated with an increased risk of heart injury. Consequently, these patients may have underlying chronic cardiovascular diseases that are asymptomatic and undetected, leading to myocardial injury and elevated cTn levels in this context of acute cerebral injury [88].

Even if these patients do not exhibit acute coronary syndrome, ECG changes and elevated cardiac enzyme levels raise the suspicion of myocardial injury and heightened cardiovascular risk. A comprehensive cardiological evaluation, including an echocardiographic examination to assess potential regional wall motion disorders, is necessary. However, significant increases in cTn levels, in conjunction with ECG changes and regional wall motion disorders, suggest the presence of acute coronary syndrome following a stroke. The acute coronary syndrome occurs in 1–3% of cases of AIS, typically within the first month and then the risk gradually decreases [89]. One potential mechanism for acute coronary syndrome is coronary atherothrombosis, which is precipitated by increased plaque instability in the context of systemic inflammation. Another mechanism involves the imbalance between myocardial oxygen demand and supply due to adrenergic overstimulation. Additionally, the patient’s cardiovascular risk factors and pre-existing cardiac conditions further contribute to the risk of developing acute coronary syndrome.

A study that included 1234 patients showed that positive cTn can also be associated with a cardioembolic cause for myocardial injury without its association with stroke severity so that it can assist in identifying the underlying mechanism of stroke [10].

## 11. Cardiac Dysfunction

Ventricular dysfunction, characterized by reduced left ventricular ejection fraction, regional wall motion abnormalities, or increased left ventricular volume, can occur as a complication following a stroke in association with myocardial ischemia, or it may occur in isolation. Conversely, ventricular dysfunction may also arise incidentally, independent of the stroke. In any case, ventricular systolic dysfunction was detected in 10–24% of patients, with 13–15% exhibiting moderate to severe dysfunction (ejection fraction < 40%) and 5–18% presenting with symptoms secondary to heart failure [90]. Approximately 60% of patients exhibited left ventricular diastolic dysfunction [91]. The severity of ventricular dysfunction was more pronounced in elderly patients with cardiovascular comorbidities, elevated levels of cTn, and more severe AIS [87,92,93]. A lesion in the right insula or left parietal cortex can lead to left ventricular systolic dysfunction, even in the absence of pre-existing cardiac damage [54].

Left ventricular dysfunction is a negative predictor of AIS size, degree of disability, and 90-day outcomes [91,94]. Mortality is 2.5 times higher in patients with ventricular dysfunction compared with those without it [95]. A study involving 14,000 stroke patients found that those with left ventricular ejection fraction (LVEF) < 60% had a higher short-term all-cause mortality risk compared with those with LVEF > 60%. This risk increased progressively with a further decrease in LVEF levels [96]. This study indicated that even an LVEF between 50–60%, while still within the normal range, can be associated with a more reserved prognosis.

A decrease in LVEF also predicts a higher risk of AIS recurrence [97]. Specifically, for every 5% reduction in LVEF, the risk of stroke increases by 18% [98]. The mechanism underlying the association between systolic dysfunction and AIS prognosis is likely related to cerebral hypoperfusion and decreased cerebrovascular reactivity, compounded by increased proinflammatory and prothrombotic risk [99].

Diastolic dysfunction has also been identified as a positive predictor for the recurrence of cerebrovascular events [100]. Proposed mechanisms for this association include reduced left atrial contractility, which is linked with intracardiac stasis, an increased thromboembolic risk, and reduced exercise capacity, even in asymptomatic cases, which limits functional recovery after stroke [101]. The early initiation of neurorehabilitation is crucial for optimal recovery in neurological movement disorders, including stroke [102,103]. The brain’s plasticity is highest in the acute phase, making this period a prime opportunity to stimulate neural reorganization and functional improvement. Post-stroke cardiac complications frequently manifest acutely, thereby restricting the intensity and duration of physical therapy, a fundamental component of neurorehabilitation [104].

Brain natriuretic peptide (BNP) and N-terminal pro-B-type natriuretic peptide (NT-proBNP) serve as specific biomarkers for ventricular dysfunction. Their levels rise within the first 24 h following the onset of a stroke and remain elevated for up to 6 days thereafter [105]. The serum concentration of NT-proBNP correlates with the severity of post-stroke ventricular dysfunction, and it serves as an independent negative predictor of the NIHSS score, the extent of cerebral infarction, and the long-term prognosis of stroke patients [106,107]. An early rise in NT-proBNP levels has been identified in a study as a predictive marker for malignant or massive edema, hemorrhagic transformation, and mortality following reperfusion therapy in patients with AIS [108,109]. The primary mechanism underlying this association is thought to be atrial heart disease, which may precede AF diagnosed after stroke and elevate the risk of thromboembolism. Another potential mechanism involves the disruption of the BBB due to cerebral injury, leading to the increased production of natriuretic peptide in the brain and its easier passage through the compromised BBB into the bloodstream.

Cardioembolic stroke is primarily caused by embolization from the left atrium, with thrombi originating from AF. AF is associated with elevated NT-proBNP levels, making it a potential predictive factor for the risk of developing cardioembolic stroke [110].

Research indicates that NT-proBNP levels are generally higher in patients with subarachnoid hemorrhage (SAH) compared with those with AIS. Elevated NT-proBNP levels in SAH are correlated with the severity of bleeding and are predictive of adverse outcomes [111].

## 12. Neurogenic Stress Cardiomyopathy

Reversible cardiac dysfunction resulting from acute brain injuries is classified as neurogenic stunned myocardium [112]. This condition presents similarly to neurogenic stress cardiomyopathy (NSC), specifically takotsubo cardiomyopathy (TTC). It remains uncertain whether NSM and NSC are distinct pathological entities or variations of the same process. The terminology for myocardial dysfunction in the context of acute neurological injury is still under debate. Some experts advocate for “stressed myocardium” instead of “stunned myocardium” to highlight the catecholamine storm phenomenon observed in these patients. It is recommended to use “Takotsubo cardiomyopathy secondary to neurological disorders” and specify the type, i.e., apical, basal, midventricular, or focal.

The neurological events associated with this condition are diverse, including brain trauma, subarachnoid hemorrhage, acute stroke, epilepsy, and reversible cerebral vasoconstriction syndrome. Damage to the insular cortex is more frequently associated with neurogenic stress cardiomyopathy [113].

The incidence of NSM as a complication of cerebral events ranges from 20% to 40%, with a higher prevalence observed in cases of subarachnoid hemorrhage and brain trauma [114]. These cardiac complications are particularly prevalent in women aged between 55 and 60 years, especially those experiencing intense emotional stress [115]. NSM has been associated with an elevated risk of all-cause morbidity and mortality, as well as an increased risk of heart failure [116].

Diagnosis of neurogenic stunned myocardium (NSM) involves a combination of new ECG changes, ultrasound findings, and the temporal evolution of cardiac markers. The observed ECG changes include QT prolongation, ST segment depression, U waves, large negative T waves (cerebral type) in the precordial leads, and various arrhythmias, including life-threatening ones. Unlike ischemic changes, ECG alterations due to neurogenic stress cardiac pathology evolve over several days and typically resolve within two weeks, except for U waves or prolonged QT intervals, which may persist [116].

Ultrasound findings in neurogenic stunned myocardium (NSM) include areas of hypokinesia, akinesia, and dyskinesia of the ventricular wall. The temporal evolution of cardiac markers, such as cTn and NT-proBNP levels, is disproportionate to the magnitude and type of ECG changes observed. Unlike in acute coronary syndromes, the characteristic ascending and descending curve of serum cTn is not present in NSM [117]. In a prospective study involving over 2000 patients with acute stroke, it was observed that 13.7% of these patients exhibited elevated cTn levels [118].

## 13. Future Perspectives and Discussions

The incidence of myocardial injury among stroke survivors is notably high. Such cardiac damage following a stroke can either result in serious enduring heart conditions or lead to minor recoverable harm. Blood biomarkers and ECG evaluations are crucial for gauging the extent, prognosis, and treatment strategies for these patients, and they significantly influence clinical practices. Phase-rectified signal averaging (PRSA) is also an ECG-based method for evaluating cardiac autonomic function, which reflects sympathetic and vagal nerve activities by calculating the acceleration (AC) and deceleration (DC) capabilities of the heartrate when applied to long-term-recordings of heartbeat intervals. A reduction in DC is often associated with impaired vagal function and is considered a marker for increased cardiovascular risk. DC reduction has been observed in conditions like heart failure, diabetes, or after a myocardial infarction [119].

It is essential to determine whether these cardiac abnormalities are due to pre-existing heart disease or a direct consequence of the acute cerebrovascular event. Therefore, thorough cardiac assessments and investigations for myocardial injury are vital for identifying underlying cardiovascular issues. Since cardiovascular problems are a leading cause of mortality after an acute stroke, careful monitoring of patients is imperative. Additionally, biomarkers like NT-proBNP offer valuable insights into both immediate and long-term prognoses after a stroke, impacting clinical management.

As previously mentioned in the article, circulating microvesicles and intestinal microbiota play a role in the pathophysiology of SHS. However, their specific mechanisms and how they can be targeted to help manage SHS remain unclear, highlighting the need for further research.

The primary challenge moving forward will be to establish targeted therapeutic strategies for SHS and its long-term sequelae. Patients with acute coronary syndrome following a stroke should be prioritized for early coronary revascularization [120]. A reasonable approach for early-stage treatment of synchronous AIS and acute myocardial infarction, according to the 2019 guidelines from the AHA/American Stroke Association, is administering intravenous thrombolysis with alteplase at the therapeutic dose for AIS within 4.5 h, followed by percutaneous coronary intervention if indicated [62]. In the future, in addition to standardized electrocardiography and echocardiographic assessment, stroke patients with relevant hs-cTn elevation could routinely undergo other diagnostic measures involving contrast-enhanced multi-slice cardiac computed tomography and/or comprehensive cardiovascular magnetic resonance imaging to differentiate ischemic and non-ischemic cardiac pathologies. Cardiovascular magnetic resonance imaging may also offer superior accuracy in detecting subtle cardiac changes related to cardiac ischemic processes.

Recent research has proposed a comprehensive care model based on the ABC (Atrial fibrillation Better Care) pathway for patients with SHS that encompasses three key components: (A) antithrombotic therapy; (B) better functional and psychological status; and (C) cardiovascular risk factors’ and comorbidities’ management [121].

Antithrombotic therapy (e.g., temporary dual antiplatelet therapy or adjunctive low-dose anticoagulation) is fundamental in both conditions, but its efficacy in preventing recurrent vascular events remains uncertain. Because of the distinct mechanisms of SHS, managing symptoms and cardiovascular risk factors may include modulation of the sympathetic nervous system, heart rate regulation, and stabilization of blood pressure variability (e.g., β-blockers, ivabradine, renin–angiotensin system inhibitors), along with anti-inflammatory strategies (e.g., inhibition of inflammasomes or inflammatory cytokines such as interleukin-1β using agents like colchicine, canakinumab, or rilonacept). Additionally, enhancing vascular endothelial function, mitigating oxidative stress (e.g., with statins, renin–angiotensin system inhibitors, antioxidants, and new-generation monoamine oxidase inhibitors), and avoiding proarrhythmic drugs (such as those causing QTc prolongation) may be beneficial [52]. Edaravone may be effective in treating various acute cerebrovascular conditions and providing cardioprotection in stroke patients. Edaravone provides a cerebral neuroprotection and cardiovascular protection by reducing oxidative stress and inflammation, but its exact mechanism of action remains unknown [122,123].

Recent guidelines recommend specific targets for blood pressure, low-density lipoprotein (LDL) cholesterol, and glycated hemoglobin levels in patients with AIS; however, optimal targets for those with concurrent cardiac conditions remain undefined [124]. Moreover, cardiac manifestations arising from non-atherosclerotic etiologies necessitate comprehensive evaluation to identify the underlying cause and guide appropriate treatment. Most stroke guidelines recommend routine cardiac evaluations post-stroke, including but not limited to standard ECG, 24 h Holter monitoring, and echocardiography. At present, it is not clear whether the high frequency of systolic and diastolic dysfunction detected in stroke patients is transient and recovers over time or whether this will result in clinically manifest heart failure.

Remote ischemic conditioning (RIC) has emerged as a safe and promising intervention for cardio-cerebrovascular diseases, demonstrating protective effects through multiple mechanisms [125]. Therefore, early application of RIC following stroke onset may be beneficial in preventing the development of SHS, and providing resistance to subsequent insults. Using a manual blood pressure cuff, the standard RIC protocol consists of four cycles of 5 min of inflation at 200 mmHg, followed by 5 min of deflation, thus applying brief controlled ischemia followed by reperfusion in one vascular bed or tissue (usually a limb). These cycles of ischemia and reperfusion might confer protection to remote organs, such as the brain and heart, enhancing their resistance to ischemic damage [126].

Cannabinoid compounds such as tetrahydrocannabinol (THC) and cannabidiol (CBD) have been observed to potentially improve endothelial function, optimize cardiac perfusion, mitigate the risk of developing cardiac arrhythmias, and reduce oxidative stress [127]. Moreover, they reduce blood–brain barrier permeability, enhance cerebral blood flow, and alleviate spasticity [128,129]. However, evidence also suggests a possible association between cannabis use and an elevated risk of adverse cardiovascular events, including heart failure, coronary heart disease, and stroke [130]. Further research is essential to elucidate the therapeutic potential and differential effects of various cannabinoids across a spectrum of disease states, addressing the current gaps in our understanding.

The treatment approaches for SHS are summarized in Table 1.

## 14. Conclusions

To date, there are no established guidelines for the assessment, management, or follow-up of ischemic stroke patients who develop post-stroke cardiac injury from a cardiological standpoint. Collaborative research efforts spearheaded by interdisciplinary teams of cardiologists and neurologists, involving well-defined exclusion criteria and adequate statistical power, with both preclinical and clinical perspectives, are essential to close the knowledge gap between cardiac injury and clinical outcomes in acute stroke patients.

This narrative review is subject to several limitations. Firstly, as a narrative review, it does not include a formal quality assessment or meta-analysis of the included studies, which may lead to selection bias. The exclusion of non-English studies may also limit the diversity of perspectives and findings, as important research published in other languages may have been overlooked.

Furthermore, the focus on studies published from 2015 to 2024 means that some earlier foundational studies may not have been included, potentially omitting relevant historical data or insights. Lastly, this review does not incorporate new primary data or original research, relying solely on existing studies, which may not fully capture emerging trends or novel insights in the field. These limitations should be considered when interpreting the findings, as they may affect the generalizability and comprehensiveness of the conclusions drawn.

## Figures and Tables

**Table 1:** Treatment approaches of SHS [52,120,121,125].

Treatment Strategy	Summary
Brain surgery	Controlling bleeding, reducing intracranial pressure, and preventing further neurological damage in hemorrhagic stroke
Cerebral reperfusion therapy	Restoring cerebral blood flow in AIS, either by intravenous thrombolysis with recombinant tissue plasminogen activator or mechanical thrombectomy.
Coronary revascularization	Restoring coronary blood flow in acute coronary syndrome either by percutaneous coronary intervention or by coronary artery bypass grafting.
Antithrombotic therapy (temporary dual antiplatelet therapy or low dose anticoagulation)	Antithrombotic therapy is a cornerstone in the management of both acute coronary syndrome and ischemic stroke to prevent thrombus formation and reduce the risk of recurrent vascular events.
Autonomic dysfunction management (β-blockers, α-blockers, ivabradine, renin–angiotensin system inhibitors)	Reducing effects of sympathetic overstimulation in order to control heartrate, and improve blood pressure variability
Vascular dysfunction and oxidative stress management (statins, renin–angiotensin system inhibitors, antioxidants)	Strategies that include blood pressure control, lipid management, lifestyle modifications (regular exercise, dietary changes and smoking cessation)
Anti-inflammatory strategies (edaravone, colchicine, canakinumab, rilonacept)	Management of inflammation might improve recovery and prevent further injury by secondary brain damage in both ischemic and hemorrhagic strokes
Rehabilitation treatment	Helping stroke patients regain function, improve quality of life, and minimize disability through physical therapy, occupational therapy, speech and language therapy, spasticity management.
Remote ischemic conditioning	Using a manual blood pressure cuff, brief, controlled episodes of ischemia and reperfusion are applied to a remote tissue or organ, typically a limb.

Legend: AIS—acute ischemic stroke.

## Data Availability

Not applicable.
